# Reproductive Strategies and Embryonic Development of Autumn-Spawning Bitterling (*Acheilognathus rhombeus*) within the Mussel Host

**DOI:** 10.3390/biology13090664

**Published:** 2024-08-26

**Authors:** Hyeongsu Kim, Jongryeol Choe, Myeonghun Ko

**Affiliations:** 1 Aquaculture Research Division, National Institute of Fisheries Science, Busan 48513, Republic of Korea; kimk2k@korea.kr (H.K.); ht4560@naver.com (J.C.); 2 Kosoo Biology Institute, Seoul 07955, Republic of Korea

**Keywords:** autumn spawning, bitterling, diapause, host, mussels, embryonic development

## Abstract

**Simple Summary:**

This study explores how the autumn-spawning bitterling, *Acheilognathus rhombeus*, reproduces and develops within its mussel host. The research highlights how these fish lay their eggs inside mussels, which then serve as a safe environment for the eggs to develop over winter. A critical phase, called diapause, allows the embryos to pause their development for about seven months, ensuring they survive the cold winter months. Once the temperature rises above 10 °C, development resumes. During this time, tiny structures on the embryos called minute tubercles help anchor them within the mussel, preventing them from being expelled prematurely. These findings provide valuable insights into the complex relationship between the bitterlings and their mussel hosts, revealing important evolutionary adaptations that aid in the reproductive success and survival of these fish.

**Abstract:**

We investigated the reproductive strategies and embryonic development of *Acheilognathus rhombeus* (a bitterling species that spawns in autumn) within its freshwater mussel host in the Bongseo Stream, South Korea. By focusing on survival mechanisms during critical stages of embryonic development, the selective use of mussel gill demibranchs by the bitterlings and associated adaptive traits were observed over 1 year. A significant diapause phase occurs at developmental stage D, which lasts for approximately 7 months, allowing embryos to survive winter. Development resumes when the temperature exceeds 10 °C. Minute tubercles on the embryos (crucial for anchoring within the host gill demibranchs and preventing premature ejection) exhibit the largest height during diapause, and the height decreases when developmental stage E is reached, when growth resumes. *Acheilognathus rhombeus* embryos were observed in 30.5% of the mussels, mostly within the inner gills, thereby maximizing spatial use and oxygen access to enhance survival. These results highlight the intricate relationship between *A. rhombeus* and its mussel hosts, demonstrating the evolutionary adaptations that enhance reproductive success and survival. This study provides valuable insights into the ecological dynamics and conservation requirements of such symbiotic relationships.

## 1. Introduction

Co-evolution refers to the specialized evolution of species through interactions within various ecological networks, resulting in mutual evolutionary adaptations among different organisms and populations. This dynamic aspect is fundamental to behavioral and evolutionary ecology research because it significantly influences species differentiation and adaptive processes [1]. Co-evolution is particularly evident in mutualistic, commensal, and parasitic relationships where species interact as predators, hosts, parasites, or symbionts, thereby shaping their evolutionary paths [2,3]. The mutualistic relationship between bitterlings (Cyprinidae: Acheilognathinae) and freshwater mussels (Unionidae) serves as an exemplary model for studying evolutionary host specialization [4,5].

In oviparous fishes such as bitterlings, which lack parental care, various reproductive strategies are crucial for enhancing fertilization rates and embryo survival. These strategies range from selecting optimal spawning sites to developing physiological adaptations that support embryonic development under fluctuating environmental conditions [6]. The choice of spawning location is critical, affecting not only embryo survival but also reproductive success [7,8,9,10]. In contrast, species that do not provide parental care are more vulnerable during development, facing biological threats such as parasites, predators, and competitors, as well as abiotic stressors, including variable oxygen levels and temperatures [6].

To improve reproductive efficiency, many species employ mechanisms such as sperm storage, delayed development, and embryonic diapause [11]. Diapause is a developmental delay triggered by regular and adverse environmental conditions during certain developmental stages [12]. This physiological dormancy enables survival through severe fluctuations in the environment, such as extreme drought or food scarcity. Although diapause is predominantly observed in arthropods, particularly insects, it has also been reported in fish, particularly annual killifish and bitterlings [13,14,15]. Diapause is a strategy used to cope with egg desiccation in killifish and low temperatures in bitterlings, facilitating their survival under challenging conditions [16,17].

Bitterlings are primarily observed across Northeast Asia and Europe, where they exhibit significant ecological interactions with bivalves from the Unionidae family [18,19,20,21]. During the reproductive phase, female bitterlings use their elongated ovipositors to deposit eggs directly into the gills of mussels via exhalant siphons. Males displaying vibrant nuptial colors establish territories around these mussels. As females lay their eggs, males release sperm into the mussel via inhalant siphons, facilitating fertilization within the gill chambers. The eggs undergo a developmental period inside the mussel that lasts for approximately 1 month, during which they absorb nutrients from the yolk. Once the embryos develop into free-swimming stages, they exit the mussels and begin independent feeding [20,22].

Some studies have suggested that bitterlings and mussels exhibit a classical host–parasite dynamic relationship [23,24,25]. To manage this relationship, bitterlings employ critical oviposition strategies to minimize the risk of premature embryonic expulsion, demonstrating significant morphological and physiological adaptations for reproduction within mussel hosts [26,27,28]. A key adaptation involves the development of minute tubercles, which are single-celled epidermal structures on embryos. These tubercles anchor embryos within the mussel gills to prevent early ejection [29,30,31,32,33]. These tubercles are primarily located near the anterior part of the head, close to the eyes of the embryos, in the early developmental stages [32,34,35,36,37,38,39,40]. The morphological presence of these tubercles is hypothesized to protect embryos from premature ejection, facilitating their development within the protective environment of host mussels. Recent studies have shown that bitterling embryos undergo a unique developmental process known as blastokinesis, where they perform a “front flip” on the yolk sac to position themselves optimally for survival within the mussel gills [41]. This front flip ensures that the embryos are aligned in a head-down position, which helps them resist being expelled by the host mussel [41].

Bitterling species can be divided based on their time of spawning. Some species spawn during spring and some during autumn. Many bitterlings reproduce in spring. However, three specific species [*Acheilognathus rhombeus* (Temminck & Schlegel), *A. typus* (Bleeker), and *A. longipinnis* Regan] exhibit autumn-spawning behavior, typically between August and November [42]. These autumn-spawning species deposit their eggs within mussels during this season; the embryos overwinter until the following spring, lasting approximately 7 months. A critical adaptation of these species is their ability to extend diapause during a particular stage of embryonic development, which aids in their survival under variable environmental conditions [17]. Furthermore, the association between the development of minute tubercles in the embryonic skin and the migration of the embryos within mussels during winter has not been characterized.

This study aimed to provide a comprehensive ecological analysis of the gra employed by the autumn-spawning bitterling, *A. rhombeus*. As an ornamental fish, it is a species of interest in Japan and Korea. This study focused on the selection of host mussels and physiological adaptations that enhance survival across different developmental stages. Using various methods, we examined how they select mussels for spawning, their movement patterns within the mussels to optimize survival at different developmental stages, and the changes in the height of minute tubercles that prevent premature ejection by other fish in this subfamily. Specifically, this study investigated how *A. rhombeus* utilizes host mussels and adapts physiologically over time, highlighting the evolutionary benefits of these adaptations in boosting survival rates.

## 2. Materials and Methods

### 2.1. Study Area

This study was conducted over a 150 m segment of Bongseo Stream, part of the Mankyeong River in Wanju-gun, Jeollabuk-do, Korea (35°53′20.07″ N, 127°10′15.37″ E), from July 2019 to July 2020. This site was selected because of the presence of a large population of *A. rhombeus*. The stream’s width and depth were 5–20 m and 0.5–1.5 m (average 1.0 m), respectively. The streambed was composed of silt, mud, and abundant submerged vegetation, supporting 23 species of freshwater fish and a single mussel species, *Nodularia douglasiae*.

### 2.2. Spawning Period

From July 2019 to July 2020, biweekly sampling was conducted using a kick net with a 3 × 3 mm mesh to determine the spawning period of *A. rhombeus*. Mature females were identified by the presence of more than five mature eggs via gentle abdominal palpation. Standard and ovipositor lengths were measured with an accuracy of 0.01 mm. The length of the ovipositor was measured from its tip to the gonopore after the water was drained, and the specimens were positioned on a flat surface. The specimens were returned to the stream immediately after measurements were taken.

### 2.3. Developmental Stage of the Embryos inside Mussels

From July 2019 to July 2020, mussels were collected by hand with a kick net along bank areas with a notably high density of mussels. Water temperatures in the natural habitats were concurrently recorded. A mussel-opening device was used to safely open the mussels up to approximately 1 cm for inspection. The shell lengths of the mussels with and without embryos were measured with an accuracy of 0.01 mm. Mussels containing *A. rhombeus* embryos were immediately preserved in a 10% neutral formaldehyde solution to prevent embryo ejection. On the collection days, the developmental stages of *A. rhombeus* within the mussels were documented using a Nikon SMZ-10 stereoscopic microscope (Nikon Corporation, Tokyo, Japan) and analyzed using AxioVision LE software (version 4.5; Carl Zeiss, Oberkochen, Germany). The embryos were categorized into stages A–I from the egg stage as per the classifications of Suzuki and Jeon [43] and Kim et al. [44].

### 2.4. Observation of the Minute Tubercles on the Embryos

**Figure 1:**
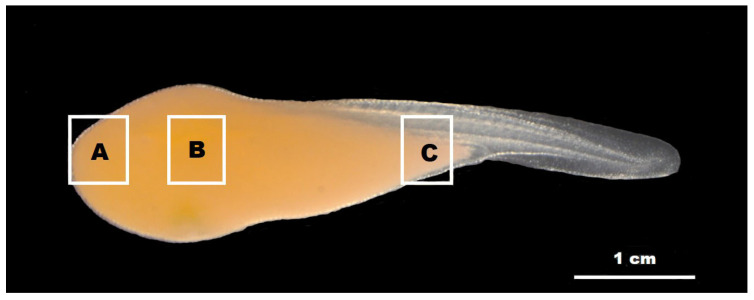
Regions of the skin surface of embryos on which the minute tubercles were distributed. (**A**), eyes and head regions; (**B**), central region of the yolk sac; (**C**), posterior region of the yolk sac.

Height variations in minute tubercles at various embryonic developmental stages were analyzed using scanning electron microscopy (SEM). Measurements were performed from the skin surface of the embryo to the apex of the tubercle. For the SEM analysis, three specimens were selected every 24 h to ensure consistent stage representation and developmental morphology variability. The specimens were fixed in cacodylate-buffered 2.5% glutaraldehyde, dehydrated using ethanol gradient, and subjected to critical-point drying with liquid CO_2_ [40]. Further, the specimens were coated with gold by ion sputtering to enhance SEM imaging (Supra40VP, Carl Zeiss, Jena, Germany). Tubercle images were captured using a Carl Zeiss vision camera (LE REL. 4.4, Jena, Germany). To assess the distribution and height of minute tubercles, the embryonic surface was divided into three sections in Figure 1: (A) the anterior region encompassing the eyes and head (EHR), (B) the central region of the yolk sac (MR), and (C) the posterior region of the yolk sac (PR). Tubercle heights across these regions were systematically measured from embryonic developmental stages A–I, with 30 samples analyzed from each region per embryo, providing a comprehensive dataset for statistical analysis.

### 2.5. Utilization of Host Mussels

**Figure 2:**
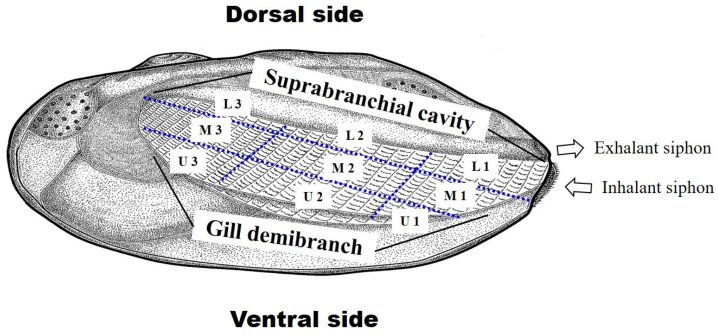
Gill demibranch and suprabranchial cavity of *Nodularia douglasiae*. The arrows indicate the general flow of water currents entering via the inhalant siphon and exiting via the suprabranchial cavity and exhalant siphon. U: upper part; M: middle part; L: lower part. Blue dotted lines: nine section in the gill demibranch.

The mussel *N. douglasiae* possesses four gill demibranchs (left, right, outer, and inner), within which mussel larvae (termed glochidia) were first observed on 16 March 2020. To analyze the positioning of *A. rhombeus* embryos within the gills, the gills were segmented into nine sections. These sections included three divisions from the suprabranchial cavity to its point of contact with the gill demibranch: lower (L), middle (M), and upper (U). Additionally, each gill was divided into three zones extending outwards from the mussel’s siphon, with zone 1 being the farthest from the outer edge (Figure 2). The precise position of the embryos within the mussels was captured and verified using a Canon Mark III camera (Tokyo, Japan). Measurements were performed to record the longitudinal distances from the suprabranchial cavity and transverse distances from the siphon to the gill demibranchs. The developmental characteristics of *A. rhombeus* embryos were further examined using a Nikon SMZ-11 stereoscopic microscope (Nikon Corporation, Tokyo, Japan) and analyzed using AxioVision LE software (version 4.4, Carl Zeiss, Jena, Germany).

### 2.6. Identification of the Species of Bitterling Embryos

Six sympatric bitterling species were identified in the study area: *Rhodeus notatus*, *R. uyekii*, *R. ocellatus*, *A. chankaensis*, *A. lanceolatus*, and *A. rhombeus*. All bitterling species except *A. rhombeus* spawned from April to July. Therefore, embryos observed within the mussels from September to March were considered to be *A. rhombeus*, and their numbers and positions were systematically recorded. The first sightings of spawned embryos of *R. notatus*, *R. uyekii*, and *A. lanceolatus* occurred on 13 April 2020, whereas those of spawned embryos of *R. ocellatus* and *A. chankaensis* occurred on 27 April 2020. *Acheilognathus rhombeus* embryos are distinguishable from those of other bitterlings by the formation of optic cups at the developmental stages D and E. Furthermore, *A. rhombeus* continues to be identifiable from other species throughout their rapid developmental stages. Additionally, the developmental stages from eggs to larvae for all six bitterling species were directly compared via observation after placing them in Petri dishes.

### 2.7. Statistical Analysis

All statistical analyses were performed using the SPSS software (version 24; SPSS Inc., Chicago, IL, USA). Normality was assessed using the Fisher exact test, and homogeneity of variances was checked using Levene’s test. The Kruskal–Wallis H test was used to evaluate variations in the number of embryos and their frequency of appearance across different gill positions in mussels. A *p*-value < 0.05 was considered significant.

## 3. Results

### 3.1. Spawning Period

**Figure 3:**
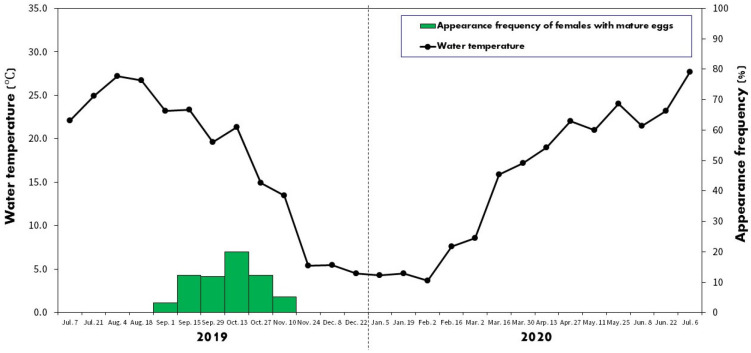
Seasonal change in the water temperature and appearance frequency of *Acheilognathus rhombeus* females in the study area.

Figure 3 shows the variations in water temperature in the study area from July 2019 to July 2020, highlighting the seasonal changes affecting embryo development. The lowest water temperature recorded was 3.7 °C on 2 February 2020, increasing after 16 February 2020. In total, 31 female *A. rhombeus* bitterlings with fully developed eggs were observed in the study. Their average standard length and ovipositor length were 61.8 ± 4.5 mm (range 52.1–71.4 mm) and 32.3 ± 4.1 mm (range 26.2–40.4 mm), respectively. On average, each female contained approximately 33.6 ± 15.9 eggs (range 14–78 eggs).

During the spawning season in 2019, the number of females with mature eggs was recorded as follows: one female (3.2% of all captured females) on 1 September, five females (12.2%) on 15 September, seven females (11.9%) on 29 September, eleven females (20.0%) on 13 October, five females (12.2%) on 27 October, and two females (5.1%) on 10 November. No females with mature eggs were observed from 24 November 2019 to 6 July 2020.

### 3.2. Developmental Stages of the Embryos inside Mussels

**Figure 4:**
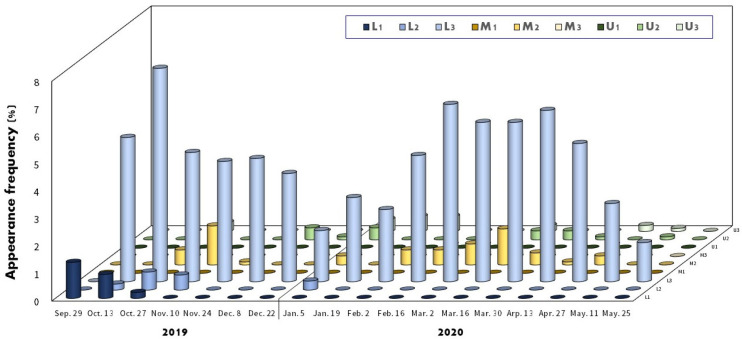
Appearance frequency of *A. rhombeus* embryos in eight parts of gill demibranch inside the mussel. The color bars indicate the position of U1, U2, U3, M1, M2, M3, L1, L2, and L3, respectively.

The embryonic developmental stages of *A. rhombeus* within host mussels are documented in Table 1 and Figure 4. From 29 September 2019 to 25 May 2020, 914 embryos were observed. Developmental delays began at stage C, progressed to diapause at stage D, and lasted approximately 3 months. Development resumed on 30 March 2020, with embryos advancing to stage E as water temperatures increased to 8–15 °C.

Eggs were first detected on 29 September 2019, when the water temperature reached an optimal value of 19.6 °C, and their presence declined after 27 October 2019. Significant developmental milestones were recorded, with the highest percentage of embryos at various stages: 100.0% at the egg stage on 29 September, 29.2% at stage A, 50.6% at stage B on 27 October, and 48.8% at stage C on 22 December 2019. No eggs, stage-A embryos, or stage-B embryos were recorded on 10 November 2019. Stage-C embryos were observed from 13 October 2019 to 19 January 2020, and stage-D embryos were observed from 24 November 2019 to 13 April 2020, with stage-D embryos exclusively present from 2 February to 16 March 2020.

By 13 April 2020, the proportion of embryos at stage E increased to 72.6%, with 27.4% remaining at stage D. On 27 April 2020, the embryos reached stages F (50.9%) and G (21.8%), marking the end of stage D. Most embryos progressed to stages G (83.3%) and H (6.7%) by 11 May 2020, with no stage-I embryos detected. Additionally, eggs of sympatric bitterling species (*A. lanceolatus*, *R. uyekii*, and *R. notatus*) were first observed on 13 April 2020. *R. ocellatus* and *A. chankaensis* eggs were detected on 27 April 2020, after the reactivation of *A. rhombeus* larval development.

### 3.3. Observation of the Minute Tubercles on the Embryos

**Figure 5:**
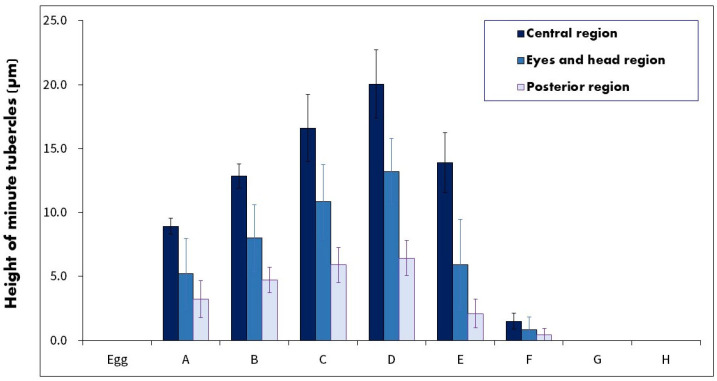
Height of minute tubercles on the surface of three regions of the yok sac in *A. rhombeus* embryos, expressed as the mean ± standard deviation (SD).

**Figure 6:**
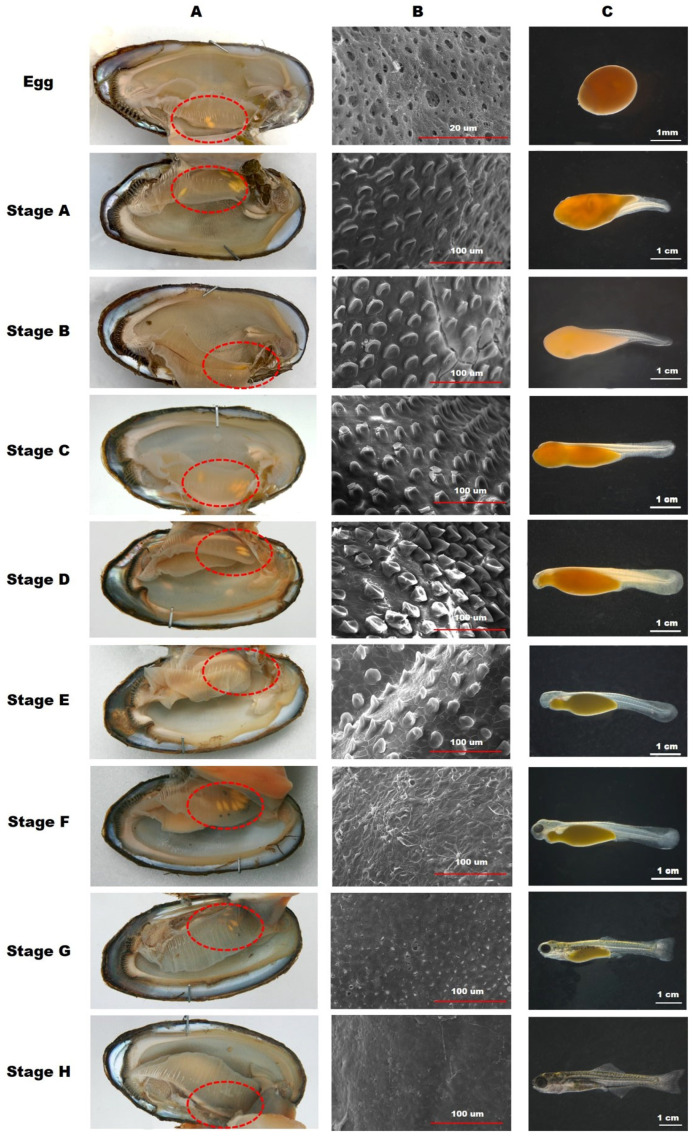
Position of *A. rhombeus* embryos inside the mussels. (**A**), development of minute tubercles on the skin surface; (**B**), and morphological characteristics; (**C**), after the embryonic development stage. The dotted circle indicates the position of embryos inside mussels.

The minute tubercles on the embryos were examined in three regions: EHR, MR, and PR. Their heights are shown in Figure 5 and Figure 6. The tubercles, which were hemispherical, appeared immediately at stage A in all three regions. The height of tubercles in the MR was as follows: 0 µm in the egg stage, 8.9 µm (4.1–15.0 µm) in stage A, 12.8 µm (7.0–19.0 µm) in stage B, 16.6 µm (10.5–22.4 µm) in stage C, 20.1 µm (15.4–24.3 µm) in stage D, 13.9 µm (6.0–21.0 µm) in stage E, 1.5 µm (0–4.0 µm) in stage F, and 0 µm in stage G and H.

Tubercle heights gradually increased from stages A–D, peaking at stage D (approximately three times the height observed at stage A), and mainly located on all surface regions. From stages D to E to F, the minute tubercle height consistently decreased by approximately 30% and 80%, respectively. At stages F, G, and H, almost no tubercles were observed in the embryonic epidermis. The tubercles were mostly hemispherical and slightly inclined from head to tail, with a higher proportion in the MR than in the HER and PR.

### 3.4. Utilization of Host Mussels

**Figure 7:**
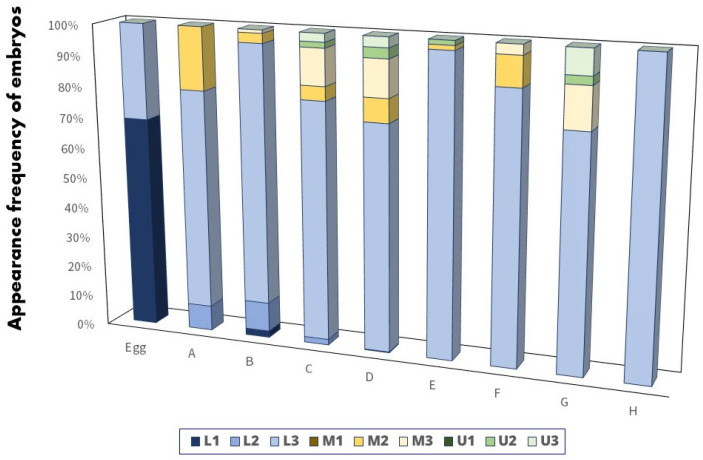
Appearance frequency of *A. rhombeus* embryos in eight parts of gill demibranch as per the embryonic developmental stage inside mussels. The color bars indicate the position of U1, U2, U3, M1, M2, M3, L1, L2, and L3, respectively.

**Figure 8:**
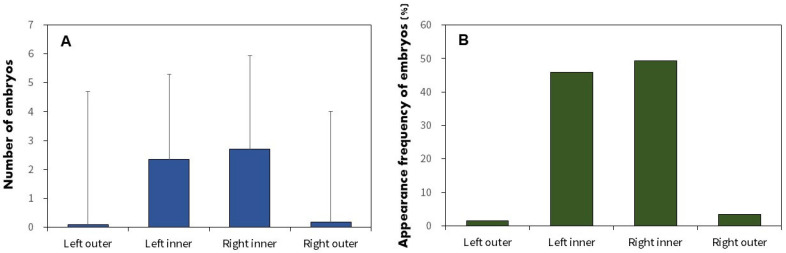
Number (**A**) and appearance frequency of (**B**) of *A. rhombeus* embryos in the four parts of the gill positions inside mussels. The values are expressed as the mean ± SD.

The distribution and position of *A. rhombeus* embryos within mussels at various developmental stages are shown in Figure 7 and Figure 8, respectively. Of the 564 collected mussels, 172 (30.5%) contained *A. rhombeus* embryos. Most embryos (80.4%; *n* = 735) were observed in the suprabranchial cavity (zones L1, L2, and L3). In contrast, only 179 (19.6%) embryos were observed in the water tube regions (M1, M2, M3, U1, U2, and U3) of mussel gill demibranchs between 29 September 2019, and 25 May 2020. The average number of *A. rhombeus* embryos per mussel was 5.3 ± 3.4 (range 1–18), based on 172 mussels.

No eggs were observed in zones M1 or U1 of the examined mussel gills. The eggs were primarily located in zones L1 (69.0%; *n* = 20) and L2 (31.0%; *n* = 9). The distribution of *A. rhombeus* embryos across various developmental stages was as follows. Stage-A embryos were mainly observed in L2 (8.2%; *n* = 4) and L3 (71.4%; *n* = 35), with some in M2 (20.4%; *n* = 10). Stage-B embryos were concentrated in L3 (84.2%; *n* = 80), with fewer in L2 (9.5%; *n* = 9), L1 (2.1%; *n* = 2), M2 (3.2%; *n* = 3), and M3 (1.1%; *n* = 1). Stage-C embryos were primarily present in L3 (77.3%; *n* = 85) and to a lesser extent in L2, M2, M3, U2, and U3. Stage-D embryos were mainly present in L3 (73.4%; *n* = 353) but were also observed in M2, M3, L2, U2, and U3.

Reactivation stages (E–H) were predominantly observed in L3. Stage-E embryos were mainly located in L3 (97.0%; *n* = 65), with sporadic occurrences in M2 and U2. Stage-F embryos were primarily observed in L3 (87.1%; *n* = 27) and M2 and M3. Stage-G embryos were notably present in L3 (75.7%; *n* = 28) and M3, U2, and U3. Stage-H embryos were exclusively observed in L3 (100%; *n* = 15).

Glochidia were exclusively located in the two outer demibranchs of the four gill types from 16 March to 6 July 2020. *Acheilognathus rhombeus* embryos displayed similar distributions across all gill demibranchs: left outer gill, left inner gill, right inner gill, and right outer gill with an average of 4.7 ± 4.6 (range 2–10; n = 14), 4.3 ± 2.9 (range 1–18; n = 395), 4.8 ± 3.5 (range 1–15; n = 477), and 4.0 ± 3.8 (range 1–10; n = 28) embryos, respectively. The Fisher exact test indicated no significant differences in embryo numbers between the left and right or inner and outer gill demibranchs (*p* > 0.05), suggesting a uniform embryo distribution within the mussel gills.

Regarding the spatial configuration of the embryos, 143 and 29 mussels contained *A. rhombeus* embryos in one and two gills, respectively. No mussel contained *A. rhombeus* embryos in three or four gills. The frequency of embryos in each of the four gill parts was 1.5% in the left outer gill (*n* = 3), 45.8% in the left inner gill (*n* = 92), 49.3% in the right inner gill (*n* = 99), and 3.5% in the right outer gill (*n* = 12; Figure 8B). Embryos were significantly more prevalent in the two inner parts than in the outer parts of the gill demibranchs, although no differences were observed among the four gill parts (Fisher exact test; *p* < 0.001).

## 4. Discussion

### 4.1. Spawning Characteristics and Embryonic Development inside Mussels

Between September and November 2019, mature eggs and early developmental stages (A and B) of *A. rhombeus* were consistently observed, corresponding with seasonal water temperatures of 13–23 °C. These conditions, which are essential for embryonic development, align with the spawning season documented by Nakamura [42]. Our results support those of Uehara et al. [16], who emphasized the critical role of temperature in initiating spawning. This reinforces the significance of water temperature not only for spawning initiation but also for influencing developmental rates and survival strategies, such as embryonic diapause onset.

The peak occurrence of spawning females was noted between 15 September and 27 October with water temperatures of 14.9–23.3 °C. This range is close to the temperature range for optimal fertilization (15–25 °C) for autumn-spawning *A. longipinnis* populations in Japan [16]. Temperature is a crucial factor affecting the developmental rate, mortality, and survival of fish embryos [45]. According to Kitamura [7], the ideal spawning conditions for bitterlings involved balancing fluctuating temperatures, which affects embryonic growth rates and oxygen levels. Unlike spring-spawning bitterlings, which reproduce between April and July, with peak activities in late April and mid-May at temperatures of 15–25 °C, *A. rhombeus* require maintaining water temperatures at approximately 20 °C during its spawning period to ensure optimal embryonic survival before temperatures decline rapidly.

Our observations indicate that *A. rhombeus* embryos in Korea take approximately 8 months, from September 2019 to May 2020, to progress from hatching to the free-swimming stage (stages A–I). Embryonic diapause occurred prior to eye pigmentation at stage D (Figure 3 and Figure 4). These findings are consistent with those of previous field studies and controlled-rearing experiments [15,42,44]. Conversely, Suzuki and Jeon [43] reported a 2-month retardation in the development of Korean *A. rhombeus* without a diapause phase. Nakamura [41] attributed these developmental delays to low temperatures, whereas Kawamura and Uehara [15] and Suzuki and Jeon [43] suggested a significant genetic influence. Notably, during the cold period from mid-November 2014 to mid-February 2019, when water temperatures were 4–5 °C, embryos remained at stage C for 3 months but advanced to stage D within 2 days as temperatures increased to approximately 22 ± 1 °C. These findings suggest that both environmental and genetic factors regulate embryonic development and diapause in *A. rhombeus*. Significant physiological differences observed across different developmental stages suggest potential subspecific variation, highlighting the need for further genetic and morphological studies to explore adaptive evolutionary strategies within this species across diverse ecological conditions [46].

On 30 March 2020, the reactivation from embryonic diapause (stage E) was observed in *A. rhombeus* as the water temperature increased to 17.2 °C. This increase in temperature (Figure 2 and Figure 3) facilitated the resumption of embryonic development. Denlinger and Armbruster [47] reported that diapause termination was often triggered by intracellular responses to environmental stimuli such as temperature increases. Additionally, studies by Gerisch and Antebi [48] in invertebrates and by Denlinger and Armbruster [47] in mosquitoes demonstrated that cholesterol and its derivatives, along with the interaction between temperature and photoperiod, play crucial roles in ending diapause. Compared with other spring-spawning bitterlings such as *A. lanceolatus*, *R. uyekii*, *R. notatus*, *R. ocellatus*, and *A. chankaensis*, the earlier reactivation of *A. rhombeus* embryo development suggested strong evolutionary pressure to optimize survival chances in response to a blend of biotic and abiotic factors [7,49,50]. Although a water temperature of 10 °C is suboptimal, it serves as a stimulatory threshold for the resumption of embryo development in bitterlings. These unique developmental adaptations enable *A. rhombeus* embryos to reach the free-swimming stage and leave their mussel hosts earlier than those of other sympatric species, thereby reducing density-dependent mortality during the peak spawning period [44].

### 4.2. Changes in the Minute Tubercles on Embryos

In *A. rhombeus* embryos, the form of the yolk projection and type of minute tubercles were categorized into B and two types with similar morphologies, such as *A. longipinnis*, *A. macropterus*, *A. chankaensis*, and *Pseudoperilampus typus* [38]. Comparative analysis within *Acheilognathus* revealed different morphologies. *Acheilognathus limbata*, *A. somjinensis*, *A. signifer*, and *A. lanceolatus* exhibited both hemispheric or scaly shapes resembling circular cones and vestigial shapes. Conversely, among *Rhodeus* species, *R. ocellatus*, *R. suigensis*, *R. atremius*, and *R. ocellatus smithi* predominantly displayed hemispheric shapes, whereas *R. pseudosericeus* and *R. uyekii* exhibited both forms [29,32,33,35,36,51,52]. These structures—particularly the unique minute tubercles and wing-like projections known to aid in preventing the premature ejection of embryos from mussel hosts—are notably absent in genera such as *Acheilognathus* (*Tanakia*). This distinction highlights the role of minute tubercles as an evolutionary adaptation unique to the *Rhodeus* genus within the bitterling family, reflecting a specialized ecological interaction with their mussel hosts. The development and diversification of minute tubercles across bitterling species represent a critical evolutionary biological trait characteristic of this fish family, highlighting the complex interplay of morphological evolution driven by ecological needs.

In *A. rhombeus*, minute tubercles exhibit hemispherical shapes and are approximately 20% taller in MR than in the EHR of the embryos. The tubercles are slightly inclined from the head towards the tail, resembling a harpoon mechanism that facilitates adherence to the gill demibranchs. This minimizes the risk of premature ejection, a common characteristic in bitterling embryos [20]. The tubercles on the MR reach their maximum height immediately post-hatching but exhibit a significant size reduction when stage E is reached, coinciding with the time of reactivation in embryonic development. Embryos are strategically positioned through the mussel’s exhalant siphon into the gill demibranchs, settling predominantly in the interlamellar spaces, where the MR offers the largest surface area [53,54]. This optimal placement was evidenced by a higher frequency (80.4%) of *A. rhombeus* embryos in the suprabranchial cavities than in the water tubes. Such positioning is likely an evolutionary adaptation to maximize oxygen acquisition and spatial benefits, ensuring a lower risk of premature displacement and enhanced survival prospects during critical developmental phases.

In the bitterlings of *Acheilognathus* (*Tanakia*), which lack wing-like projections, minute tubercles predominantly develop in the MR of the embryos. These tubercles exhibit a hilly or scaly morphology. This is distinctly different from the morphology of the tubercles of *Rhodeus* bitterlings, in which the tubercles were typically broader because of the presence of wing-like extensions [29,34,35,36,37,38,39,40,43]. Minute tubercles in *Acheilognathus* (*Tanakia*) have been adapted to ensure a firmer grip within the host mussel’s gill demibranchs, effectively reducing the risk of premature ejection. These epidermal structures were sharper and taller (20–40 µm) than those in *Rhodeus* (3–15 µm), providing tighter fit in the interlamellar spaces of mussels [27,31,55]. The development and diversification of minute tubercles across bitterling species represent a critical evolutionary biological trait characteristic of this fish family, highlighting the complex interplay of morphological evolution driven by ecological needs. Additionally, the development of these adaptations, including the front flip during blastokinesis, showcases how early developmental changes can lead to significant survival advantages in a co-evolutionary arms race with their hosts [41].

### 4.3. Distribution and Position of the Embryos in Mussels

Mussels are equipped with two distinct siphons: the exhalant siphon (which opens into the suprabranchial cavity internally connected to a water tube) and the inhalant siphon (which serves as an entry point for the external environment) [56]. Female bitterlings, particularly those with extended ovipositors, such as *A. yamatsutae*, utilize these anatomical features to deposit eggs deep within the suprabranchial cavity via the exhalant siphon, directly bypassing the water tube [53]. *Acheilognathus signifer*, *R. sericeus*, and *R. pseudosericeus* initially position their embryos in the interlamellar spaces of gill demibranchs. As these embryos develop locomotive capabilities, they migrate towards the suprabranchial cavity, moving in a direction opposite to the flow of the exhalant siphon [26,32,33,52]. Notably, *A. rhombeus* exhibits a unique embryonic placement strategy and egg shape by initially depositing eggs in the suprabranchial cavity and aligning the developmental orientation of embryos head-to-head against the exhalant siphon direction, possibly to maximize developmental advantages [44,57]. This strategic placement within the suprabranchial cavity is hypothesized to confer several benefits. It provides larger space and higher oxygen content than a narrower water tube, minimizes the energy expenditure required for post-hatching movement, reduces competition with mussel larvae (glochidia) for space and resources, and accelerates embryonic development. These adaptations enable earlier hatching and habitat settlement, offering *A. rhombeus* a developmental advantage over other sympatric species during peak spawning periods [25,27,58].

Previous research has highlighted variations in mussel gill structure and conditions, such as size, water flow speed, and dissolved oxygen content, which significantly differed across demibranch positions, between sexes, and in relation to embryo density [7,8,20,22,23,27,28,56]. In our study, *A. rhombeus* embryos are predominantly observed within the two inner demibranchs of the four gills, irrespective of the sex of the mussel. Conversely, mussel larvae (glochidia) are exclusively located within the two outer demibranchs, despite some mussel species hosting glochidia across both the inner and outer demibranchs. This spatial segregation likely reflects the embryo’s adaptive strategies to mitigate the competition and predation risks posed by glochidia, which could potentially deplete oxygen supplies or physically harm embryos through attachment [23,26]. Aldridge [26] and Mills and Reynolds [23] suggested that bitterlings preferentially utilize inner gill demibranchs over outer demibranchs because of factors such as active selection for optimal environmental conditions, greater space availability, easier ovipositor access, and reduced risk of embryo ejection. Notably, though *A. rhombeus* females can deposit eggs in female mussels devoid of glochidia, the embryos must survive from winter until early spring, often undergoing diapause. This necessitates a spawning strategy that minimizes competition for oxygen and space by leveraging the larger and presumably more hospitable environment of the inner demibranchs. Consequently, this strategic use of mussel anatomy enables *A. rhombeus* embryos to endure winter, emerging in early spring when the conditions become conducive for continued development and survival.

*A. rhombeus* demonstrates a distinct preference for spawning within the outer gill demibranchs of mussels, in contrast to the commonly observed selection of inner demibranchs by other bitterling species. This preference can be attributed to the unique spatial and environmental conditions offered by the outer demibranchs, which may better align with the specific developmental needs of *A. rhombeus* embryos. Moreover, the suprabranchial cavity is identified as the primary site for embryo deposition and is favored over the water tube because of its larger space and better conditions for oxygen availability and embryo development. This choice emphasizes the strategic adaptation of the species to maximize reproductive success in a competitive and dynamic ecological niche [59,60].

This study investigated the association between intra-clam migration and the development of epidermal protuberances during the winter in *N. japonica*. However, it did not explore molecular physiology, such as the genetic factors that induce dormancy or resume development. Identifying distinct genetic factors and triggers would greatly benefit the conservation of this species. Given that our study primarily focused on identifying the principal environmental triggers that initiate or terminate developmental dormancy in *A. rhombeus* embryos, we advocate for more comprehensive physiological studies to explore the broader effects of water temperature, photoperiodicity, and genetic influences on the mechanisms of dormancy and reactivation in this species. Such studies will provide deeper insights into the adaptive evolution of dormancy strategies among bitterlings and enhance our understanding of their ecological resilience and reproductive success under fluctuating environmental conditions. Specifically, this knowledge is crucial for developing conservation strategies for endangered species and can be applied to determine optimal conditions for artificial breeding of *Acheilognathus rhombeus* in aquaculture settings.

## 5. Conclusions

*Acheilognathsu rhombeus* embryos in mussel hosts were studied for their adaptive strategies under fluctuating environmental conditions to maintain embryonic development. A significant diapause phase, lasting approximately 7 months, occurs at the developmental stage D. It allows embryos to survive winter. At this stage, minute tubercles exhibit the largest size, aiding in anchoring within the host gill demibranchs and preventing premature ejection. When the temperature exceeds 10 °C, the growth resumes, and the height of minute tubercles decreases. *Acheilognathus rhombeus* embryos were mostly observed within the inner gills, thereby maximizing spatial use and oxygen access to enhance survival. Our research highlights the specialized adaptations of *A. rhombeus* in Korea, which enhance embryo survival within host mussels during challenging low water temperatures in winter. These adaptations are crucial for mitigating the risks of competition, exhibiting a sophisticated ecological strategy suited to unique environmental conditions.

## Figures and Tables

**Table 1:** Embryonic development stages and characteristics of embryos and minute tubercles of *Acheilognathus rhombeus* from Korea ([43,44], present study).

Embryonic Development	Stage	Characteristics of Embryos and Minute Tubercles
	Eggs	Not hatching, nearly ovoid-shaped with yellow in color No minute tubercles on the skin surface
	A	Motionless and undeveloped body parts Slight minute tubercles were observed on the skin surface
	B	The larvae tail elongated backwards, and caudal portion appeared Minute tubercles developed over the entire body
Retarding stage	C	Optic cups without lens were clearly observed and moved faster The growth of minute tubercles was observed on the head and body regions
Diapausing stage	D	The heart pulsated and notochord began to bend upwards The height of minute tubercles reached the maximum
Reactivation stage	E	Melanin pigments formed on the optic cups and moved in a wiggly pattern The height of minute tubercles on the skin surface were reduced
Eye pigmentation stage	F	Lenses were completely developed; melanin pigmentation increased The minute tubercles almost disappeared
	G	Formation of all the caudal fin rays was complete, and the dorsal and anal fin rays were formed The minute tubercles disappeared
	H	The length of the upper and lower jaws was equal; larvae started to swim slightly
Free-swimming stage	I	Larvae swam out from the mussels and began to feed

## Data Availability

The data underlying this article are available in Figshare (https://figshare.com/articles/dataset/dx_doi_org_10_6084_m9_figshare_6025748/6025748, accessed on 20 March 2024).
